# P-1407. Beyond the Patient: Educational Disruption and Household Poverty in the Wake of Tuberculosis

**DOI:** 10.1093/ofid/ofaf695.1594

**Published:** 2026-01-11

**Authors:** Sadie Cowan, Komala Ezhumalai, Senbagavalli Prakash Babu, Madolyn Dauphinais, Mandar Paradkar, Padmini Salgame, Amita Gupta, Devasahayam J Christopher, Kamakshi Prudhula, Shri Vijay Bala Yogendra Shivakumar, Perumal K Bhavani, Balamugesh Thangakunam, Vidya Mave, Sanjay Gaikwad, Jerrold J Ellner, Vijay Viswanathan, C Robert Horsburgh, Robert C Bollinger, Valarie Lyngdoh, Ashutosh Aggarwal, Sonali Sarkar, Pranay Sinha

**Affiliations:** Boston Medical Center, Somerville, MA; Dept. of Preventive and Social Medicine, JIPMER, Puducherry, Puducherry, Puducherry, India; Dept. of Preventive and Social Medicine, JIPMER, Puducherry, Puducherry, Puducherry, India; Boson Medical Center, Boston, Massachusetts; yramjee Jeejeebhoy Government Medical College Clinical Research Site (BJGMC-CRS), Pune, Maharashtra, India; Rutgers University-NJMS, Newark, New Jersey; Johns Hopkins, Baltimore, MD; Christian Medical College, Vellore, Ranipet district, Tamil Nadu, India; Bhagwan Mahavir Medical Research Centre- BMMRC, Hyderabad, Telangana, India; ICMR-National Institute of Epidemiology, Chennai, Tamil Nadu, India; National Institute for Research in Tuberculosis, Chennai, Tamil Nadu, India; Christian Medical College and Hospital, Vellore, India, Vellore, Tamil Nadu, India; Johns Hopkins University, Baltimore, Maryland; Byramjee Jeejeebhoy Government Medical College and Sassoon General Hospitals, Pune, Maharashtra, India; Rutgers New Jersey Medical School, New Brunswick, New Jersey; Prof. M. Viswanathan Diabetes Research Centre, Chennai, Tamil Nadu, India; Boston University, Boston, Massachusetts; Johns Hopkins , MD; North Eastern Indira Gandhi Regional Institute of Health and Medical Sciences, Mawdiangdiang Shillong, Meghalaya, India; Postgraduate Institute of Medical Education and Research, Chandigarh, Chandigarh, India; Dept. of Preventive and Social Medicine, JIPMER, Puducherry, Puducherry, Puducherry, India; Boston University, Boston, Massachusetts

## Abstract

**Background:**

Tuberculosis (TB), a leading cause of global mortality, disproportionately affects low socioeconomic households and deepens poverty. In India, persons with TB (PWTB) and their families face disrupted education and reduced income, further limiting access to care. TB must be recognized not just as a clinical condition but as a social disease—intertwined with education, nutrition, and economic stability—if elimination goals are to be met.

Household Income Over Course of Treatment by Education Discontinuation Status

**Figure 1.**
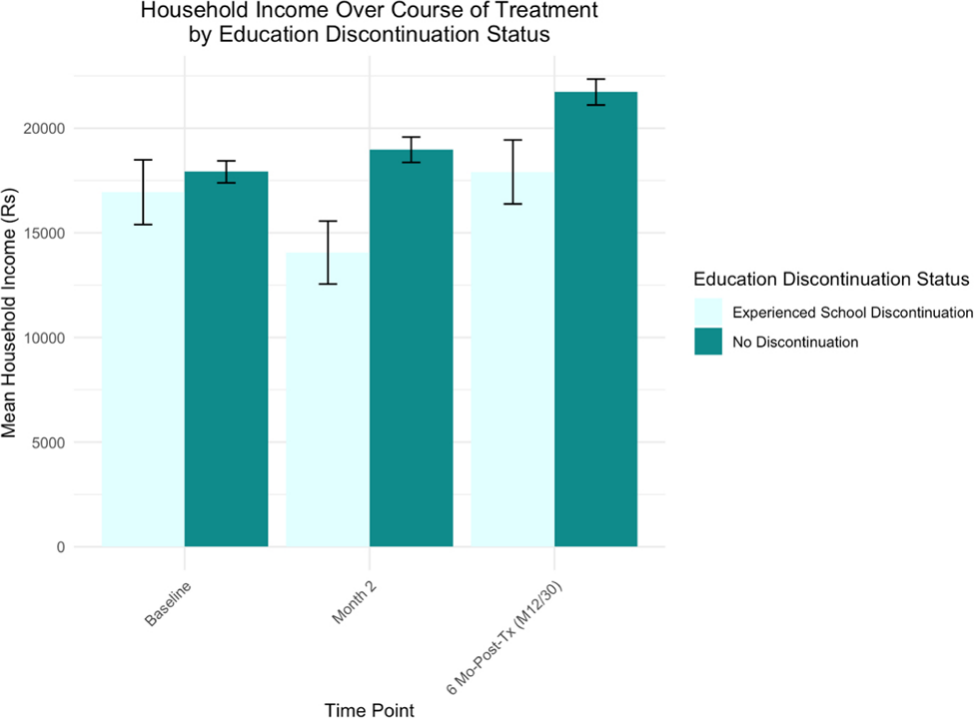
Comparison of household income over course of TB treatment between households that have at least one individual who experienced educational disruption versus the control group. Multidimensional Poverty Index Score Comparison Based on Education Status

**Figure 2.**
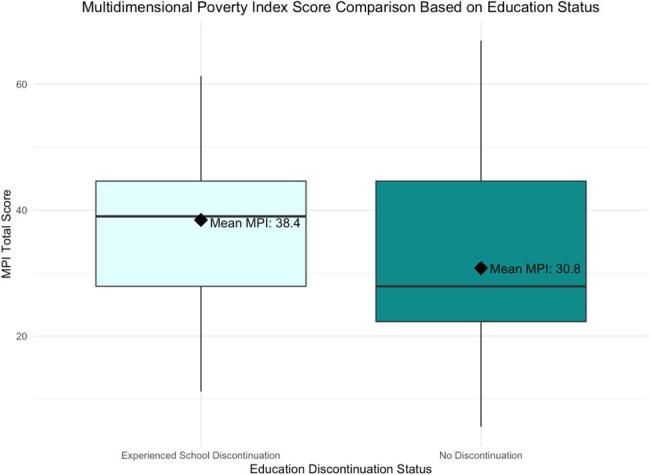
Comparison of Multidimensional Poverty Index score between households that have at least one individual who experienced interrupted schooling

**Methods:**

We used data from the RePORT India cohort, which follows PWTB in Puducherry, Tamil Nadu, Maharashtra, and Telangana. Participants were assessed at baseline; months 1 and 2 (intensive phase); month 6 (treatment completion); and at 6 and 12 months post-treatment. Clinical data included symptoms, treatment history, BMI, and Karnofsky scores. Structured interviews assessed TB’s socioeconomic effects using the Multidimensional Poverty Index (MPI), which includes education, living standards, and household composition.

**Results:**

Among 663 households surveyed, 14% reported disrupted education, 85% of which involved household contacts of PWTB. Disruption often affected multiple members; 26 unique households reported educational interruption. Households without disruption showed steady income gains between months 2 and 6 post-treatment, while those with disruption exhibited fluctuating income, suggesting prolonged vulnerability. MPI scores averaged 38.4 in disrupted households vs. 30.8 in others (poverty threshold: 33).

**Conclusion:**

TB’s impact extends beyond the patient, disrupting education and eroding household resilience. Each missed year of schooling reduces lifetime earnings by ∼8%. Protecting education during TB illness is critical to achieving household stability and national TB elimination targets.

**Disclosures:**

Robert C. Bollinger, Jr., MD, MPH, [SCENE] Health: Advisor/Consultant|[SCENE] Health: Board Member|[SCENE] Health: Stocks/Bonds (Private Company)|Merck: Advisor/Consultant|miDiagnostics: Co-inventor of IP owned by Johns Hopkins University|miDiagnostics: Eligible for equity and royalty payments received by Johns Hopkins University

